# Nanotechnology and Wood Science

**DOI:** 10.3390/nano13040691

**Published:** 2023-02-10

**Authors:** Antonios N. Papadopoulos

**Affiliations:** Laboratory of Wood Chemistry and Technology, Department of Forestry and Natural Environment, International Hellenic University, GR-661 00 Drama, Greece; antpap@for.ihu.gr

Nanotechnology, in a sense, is not entirely a new concept. A large number of chemical substances or, in some cases, chemical processes have special properties at the nanoscale level. Nanotechnology is considered to be a genuinely interdisciplinary field because it encourages cooperation between researchers who previously belonged to different fields, sharing knowledge, tools, and techniques [1]. Therefore, physics, chemistry, medicine, biology, engineering, and too many relevant subsciences belong on the nanoscale level. Indeed, it could be argued that progressive developments in each of these areas for explorations on an ever-smaller scale have now come to be known under the term “nanotechnology”. It is widely known that nanotechnology is the combination of the technology on the nanoscale level. This major advantage makes nanotechnology a huge field with numerous subsections. Nanomaterials are already used for commercial purposes in industry. The commercial products which are available nowadays belong to a broad technologic area, including stain-resistant and wrinkle-free textiles, cosmetics, sunscreens, electronics, paints, and varnishes [2,3].

Nanotechnology is being developed for a great variety of applications, from medical uses to material behavior improvement, such as wood and wood products. Studies on the application of nanotechnology in wood science are mainly focused on the dimensional stability and resistance to attack by microorganisms. The main advantage of applying nanotechnology in wood science is the unique ability of the nanoparticles to penetrate deeply into wood substrates [4,5]. It is well-established that the cell wall of wood shows a porosity of molecular scale dimensions [5,6]. The small-sized nanoparticles can easily, effectively, and deeply penetrate into the wood, to alter its surface chemistry, and to improve its properties, therefore resulting in a hyper-performance product. A review on the application of nanotechnology on wood and wood products can be found elsewhere [7,8].

This Special Issue, Nanotechnology and Wood Science, provides selected examples of high-quality works and topics focusing on the latest approaches on the development and applications of nanomaterials to both solid wood and wood products to enhance their properties.

Wang et al. [9] evaluated the effect of phenol formaldehyde resin at various concentrations (15%, 20%, 25%, and 30%) on pine wood cell walls, and the degree of resin penetration was determined using confocal laser scanning microscopy (CLSM). Subsequently, the micromechanical properties of the cell walls were analyzed using the dynamic modulus mapping technique and the method of quasi-static nanoindentation. They found that phenol formaldehyde resin can penetrate deeply into the wood tissues and that the degree of penetration decreased, accompanying the increase in the penetration depth in wood. In addition, the cell-wall mechanics maintained stable as the resin concentration increased above 20%, resulting in increasing the bulking effects, such as the decreased crystallinity degree of cellulose. 

An important issue in wood composites is the relatively low thermal conductivity coefficient of the raw material, which in turn prevents the fast transfer of heat into the core of the matrix. Taghiyari et al. [10] investigated the effect of sepiolite at the nanoscale, in a mixture with commercial urea-formaldehyde resin in manufacturing oriented strand lumber and its effect on the thermal conductivity coefficient of the final board. The hardness of the final panel was measured, at different depths of penetration of the Janka ball, in order to determine how the improved conductivity affected the hardness of the final panel, since it is known that an improved thermal conductivity would ultimately be translated into a more effective polymerization of the resin. The results indicated significant increases in the thermal conductivity coefficient of sepiolite-treated panels. Taking into consideration that the issue of the amount of sepiolite used in the panels was very low, the significant increase in the hardness was attributed to the improvement in the thermal conductivity and, consequently, the more complete curing of resin.

Since wood is susceptible to mold infection in wet environments and dense mold spots not only affect the aesthetic and decorative functions of wood but also reduce its mechanical properties, Ag/TiO_2_ wood-based nanocomposites with anti-mold functions were successfully prepared via an ultrasound impregnation and vacuum impregnation [11]. Nano-Ag/TiO_2_ can form a two-stage rough structure on wooden surfaces and introduce long-chain alkanes to make the wood hydrophobic, thus destroying the moist environment in wood that allows for the mold’s survival. At the same time, Ag/TiO_2_ was deposited in the wood pores, which reduced the number and overall volume of the pores and blocked the path of mold infection. This study revealed the anti-mold mechanism of Ag/TiO_2_ wood-based nanocomposites from the perspectives of the water content and infection path and has potentially provided a feasible pathway for wood-based nanocomposites with anti-mold functions.

Marini et al. [12] made an approach to increase the performance of adhesives such as polyvinyl acetate (PVAc) or melamine-urea-formaldehyde (MUF) by means of nanoparticles in order to obtain a material with a better mechanical and environmental resistance. When applying cellulose-based nanoparticles or tannin, the concept of a circular economy is successfully implemented into the forest/wood value chain, and chances are created to develop new value chains using byproducts of forestry operations. In this study, assortments obtained from young sweet chestnut coppice stands were utilized for the preparation of single lap joint assemblies using different commercial adhesives (PVAc, MUF) and cellulose nanocrystals (CNC) and tannin as additives. The results showed that the addition of CNC and tannin to PVAc glue increased the tensile shear strength in lap joint tests presenting a promising base for future tests regarding the addition of CNC and tannin in MUF or PVAc adhesive formulations. Unfortunately, the tested bio-based additives did not reveal the same encouraging results when tested in the wet state.

Last but not least, Tamantini et al. [13] presented an article based on cellulose nano crystals (CNC) as an additive for a bio-based waterborne acrylic wood coating. The coatings were applied on European beech and Norway spruce wood in order to test the durability against a brown and white rot wood rot fungi. It was found that the addition of CNC increased viscosity, limiting the spreading of the coating into wood pores as visible after the SEM observation, which reduced the coating adhesion on the substrate. Furthermore, CNC improved the fungal resistance as seen by a reduced mass loss and FTIR spectroscopy, thanks to crosslinks’ formation, which reduced the water sorption as well.

It can be safely and definitely said that the field of nanotechnology and wood science is a fascinating one and its future looks incredibly bright. What is presented here and in the other two Editorials is a very short overview of what will happen in the near future. Laboratories worldwide conduct innovative research and new challenges, approaches, and ideas are continuously being developed, paving an exciting and interesting research future.
